# Black Holes as Laboratories: Tests of General Relativity

**DOI:** 10.1007/s10714-025-03437-7

**Published:** 2025-06-22

**Authors:** Ruth Gregory, Samaya Nissanke

**Affiliations:** 1 Department of Physics, King’s College London, University of London, Strand, London, WC2R 2LS UK; 2 Perimeter Institute, 31 Caroline Street North, Waterloo, ON N2L 2Y5 Canada; 3 GRAPPA, Anton Pannekoek Institute for Astronomy and Institute of High-Energy Physics, University of Amsterdam, Science Park 904, 1098 XH Amsterdam, The Netherlands; 4 Nikhef, Science Park 105, 1098 XG Amsterdam, The Netherlands

## Abstract

We briefly overview the case for using black holes as a discriminator for theories of gravity. The opportunities and challenges for the various observational experiments are outlined, and key questions for the community identified. This note summarises the discussion from the roundtable on the third day of *Black Holes Inside and Out*.

The past decade has seen a complete transformation in the way black holes are regarded. A decade ago, we were pretty sure that black holes existed, but it was via deductive reasoning: It is small, heavy, and non-luminous, so what else can it be? Direct evidence was missing. However, with first the gravitational wave signal from merging black holes [1], then the imaging of the black hole shadow [2], we are now in the position of having direct evidence of strongly curved near, or at, horizon geometry.

Having this data has opened the exciting possibility that we can now test General Relativity (GR), not as a correction to Newtonian Gravity, or in the very special case of the high degree of symmetry of a cosmological background, but in the strong field regime of black hole horizon. While many of us often assume that GR is right, and certainly weak field GR has been well verified [3], the conflict between GR and Quantum Theory is so profound that it is essential we step back and ask: is GR really the right theory of gravity? Much of the theoretical conflict between gravity and quantum theory arises when we are at extreme scales, however, even the relatively mild environment of an event horizon leads to quantum puzzles: do we have a horizon, a firewall, a fuzzball [4]? It is therefore essential to test GR in these strong gravity regimes, where exotic alternatives to the standard description of compact objects might reveal their signatures.

We are currently experiencing a golden age in strong-field gravity, marked by groundbreaking observations and measurements from a range of experiments, including the LIGO [5], Virgo [6], and KAGRA [7] detectors, the GRAVITY instrument [8], and ongoing Event Horizon Telescope (EHT; e.g., [9]) campaigns. This boom period is set to expand in the 2030s and 2040s with the advent of next-generation observatories, such as funded telescopes and instruments such as the Extremely Large Telescope (ELT, e.g., GRAVITY+, [10]), the Square Kilometer Array, and the Laser Interferometer Space Antenna (LISA; [11]). Additionally, proposed projects like the next-generation Event Horizon Telescope (ngEHT; e.g., [12]), Cosmic Explorer (CE; [13, 14]), and the Einstein Telescope (ET; [15, 16]) promise further advancements. These new observatories represent major investments and a multitude of ambitious experimental advances, encompassing decades of engineering, instrumentation, and cutting-edge technological breakthroughs.

Given these developments, it is crucial that the scientific community prepares to maximize the opportunities they present for testing general relativity, particularly through the analysis of black hole observations across diverse data sets. For example, as discussed below, this includes exploring theoretical, computational, and analytical approaches to gravitational wave data, along with future GRAVITY observations.

Firstly, for gravitational waves (GWs), compared to the few hundred stellar-mass binary black hole and neutron star mergers observed since 2015, we can expect to observe several hundred to thousands of binary black hole mergers per year at redshifts up to 1, starting in the early 2030s with the proposed LIGO A$\#$ and Virgo_nEXT upgrades (see e.g., [17]). With entirely new GW facilities like ET and CE, we could observe several hundred thousand binary black hole mergers per year, reaching redshifts of up to 30 and beyond [13–16]. In the case of LISA, we anticipate detecting numerous supermassive black hole mergers per year, along with extreme mass-ratio inspirals [11].

These upgrades and next-generation GW observatories represent a paradigm shift in our ability to use black hole observations to test General Relativity. Notably, they will enable: (1) exquisite precision in high signal-to-noise ratio (SNR) events, with precision physics becoming possible for the tens of binary black hole mergers per year that will have SNRs greater than 1000. (2) greater sensitivity to more parts of the gravitational waveform, whether it be the inspiral phase of heavier binary black hole mergers or the post-merger remnant for low-mass neutron star binaries. For instance, we may observe thousands of post-inspiral binary black hole mergers with SNRs exceeding 100. (3) the discovery of a population of intermediate-mass black holes. (4) a dramatic increase in the number of binary black hole mergers due to the larger volume of space being probed. (5). The potential detection of primordial black holes, particularly from binary black hole mergers observed at redshifts greater than 20.

All of these advances will enable a plethora of tests of GR. These include improving graviton mass constraints by three orders of magnitude, conducting ringdown tests of the no-hair theorem with SNRs potentially reaching 11,000 for the root-sum-square post-inspiral of binary black holes, detecting gravitational wave memory, and testing for beyond-GR polarizations during the earlier inspiral phase, e.g., [14]. We will also be able to perform parameterized consistency tests of GR, such as those using the post-Einsteinian (PPE) formalism, e.g., [18].

To meet these challenges, significant and coordinated investment is required in developing complete and accurate approximants for binary black hole (BBH) mergers, as well as other binary sources. This is urgently needed as we approach the era of next-generation GW detectors, where SNRs could reach as high as thousands. A combination of analytical relativity, numerical relativity, effective field theory and machine learning is expected to be employed for source modeling and the production of gravitational waveforms. Additionally, advances in amplitude scattering methods are likely to play an increasingly important role in theoretical modeling, e.g., [19].

Over the past several years, it has also become clear that environmental effects-whether from beyond-standard-model physics, ultra-light boson clouds, or astrophysical environments like accretion disks-can be degenerate with one another and may mimic the effects of non-GR compact object mergers. During our roundtable discussions, it was emphasised that such degeneracies could pose a significant challenge in identifying non-standard sources or environments (e.g., [20, 21]). Therefore, it is crucial to develop a systematic and comprehensive framework for producing and analysing gravitational waveform models within the context of GR, beyond-GR theories, and beyond standard model physics, while incorporating physical effects from astrophysics, such as accretion disks and finite-size effects such as the information encoded about the ultra-cold, dense matter in the case of neutron stars. To achieve this goal, we stress the need for active collaboration across multiple disciplines including theoretical and observational astrophysics, general relativity, high energy theory and phenomenology, and nuclear physics.

Moreover, this multi-disciplinary community should also focus on the identification of clear observational signatures, or “smoking guns," for new types of GW sources. These include primordial black holes, specific resonances from binary black holes in unique environments, and the tidal deformabilities of exotic compact objects.

In this context, we stress the necessity of advancing our analysis methods to handle the multitude of GW signals from both standard and exotic sources across various beyond-GR theories. For transient events like binary black hole and neutron star mergers observed with future detectors like the ET and CE, many signals will overlap, each signal with durations potentially lasting several hours - much longer than the fraction of a second to minutes that we currently observe. Moreover, new algorithms will be essential to address the significant computational challenges involved in analyzing continuous sources, such as axisymmetric rotating neutron stars and stochastic astrophysical or cosmological backgrounds. These methods must also ensure the detection of anomalous signals, which could originate from either new sources or new physics. This will be critical in the era of next-generation GW observatories, where strong synergies will exist between space-based detectors like LISA and ground-based detectors like ET and CE. For example, in the case of LISA, preparations are already underway to implement a “global fit" for analyzing synthetic datasets spanning years (e.g., [22]), which will include signals from several supermassive black hole mergers, millions of Galactic white dwarf binary inspirals, stellar-mass black hole inspirals, and extreme mass-ratio inspirals. We are already witnessing the deployment of machine learning and artificial intelligence methods in both GW detection and parameter inference-such as simulation-based inference using convolutional neural networks (e.g., [23, 24])-as well as in the forward modeling of GW emission from various sources. These techniques are becoming increasingly critical as we prepare for the data deluge expected from future detectors, and also for successful follow-up by other electromagnetic and astroparticle telescopes for nearby golden events.

Secondly, in the case of the GRAVITY+ experiment, before addressing beyond-GR effects, there is an urgent need to account for the stronger foreground of Newtonian perturbations from other classical objects, such as stellar-mass black holes, neutron stars, and white dwarfs, near Sagittarius A*. How many of these classical objects is tied to the star formation history of the universe, as well as to how Sagittarius A* acquired its own mass. Fully modeling the astrophysical environment, while also incorporating beyond-GR effects, remains a significant challenge. Disentangling these effects will require considerable effort in both modeling and simulations, collaborations with observers and instrumentalists, especially as the precision of our data increases dramatically with the arrival of the ELT.

Finally, we must be prepared for the possibility that our current and next-generation detectors may not detect or reveal any hints of “new" physics. In that case, we must ask ourselves: what are the implications of black hole measurements for testing GR and beyond-standard-model physics? How do we interpret the absence of new physics in these observations, and what would this mean for future facilities and for our understanding of fundamental physics?

## Data Availability

No datasets were generated or analysed during the current study.

## References

[1] Abbott, B.P., et al.: Observation of gravitational waves from a binary black hole merger. Phys. Rev. Lett. **116**(6), 061102 (2016). 10.1103/PhysRevLett.116.061102. arXiv:1602.03837 [gr-qc] 26918975 10.1103/PhysRevLett.116.061102

[2] Akiyama, K., et al.: First M87 event horizon telescope results. I. The shadow of the supermassive black hole. Astrophys. J. Lett. **875**, 1 (2019). 10.3847/2041-8213/ab0ec7. arXiv:1906.11238 [astro-ph.GA]

[3] Will, C.M.: The confrontation between general relativity and experiment. Living Rev. Relativ. **17**, 4 (2014). arXiv:1403.7377 [gr-qc] 28179848 10.12942/lrr-2014-4 PMC5255900

[4] Almheiri, A., Marolf, D., Polchinski, J., Sully, J.: Black holes: complementarity or firewalls? JHEP **02**, 062 (2013). 10.1007/JHEP02(2013)062. arXiv:1207.3123 [hep-th]

[5] The LIGO scientific collaboration, Aasi, J., et al.: Advanced LIGO. Class. Quantum Grav. 32(7), 074001 (2015) 10.1088/0264-9381/32/7/074001

[6] Acernese, F., Agathos, M., Agatsuma, K., et al.: Advanced virgo: a second-generation interferometric gravitational wave detector. Class. Quantum Grav. **32**(2), 024001 (2014). 10.1088/0264-9381/32/2/024001

[7] Akutsu, T., Ando, M., Arai, K., et al.: Overview of KAGRA: detector design and construction history. Prog. Theor. Exp. Phys. **2021**(5), 05–101 (2020). 10.1093/ptep/ptaa125

[8] Gravity Collaboration, Abuter, R., et al.: First light for GRAVITY: Phase referencing optical interferometry for the very large telescope interferometer. Astron. Astrophys. **602**, 94 (2017) 10.1051/0004-6361/201730838arXiv:1705.02345 [astro-ph.IM]

[9] Event horizon telescope collaboration, Akiyama, K., et al.: First M87 event horizon telescope results. II. array and instrumentation. Astrophys. J. Lett. **875**(1), 2 (2019) 10.3847/2041-8213/ab0c96arXiv:1906.11239 [astro-ph.IM]

[10] Gravity+ Collaboration, Abuter, R., et al.: The GRAVITY+ Project: towards All-sky, Faint-Science, High-Contrast Near-Infrared Interferometry at the VLTI. The Messenger **189**, 17–22 (2022) 10.18727/0722-6691/5285arXiv:2301.08071 [astro-ph.IM]

[11] Colpi, M., et al.: LISA definition study report. arXiv e-prints (2024) arXiv:2402.07571 [astro-ph.CO]

[12] Ayzenberg, D., Blackburn, L., Brito, R., Britzen, S., Broderick, A.E., Carballo-Rubio, R., Cardoso, V., Chael, A., Chatterjee, K., Chen, Y., Cunha, P.V.P., Davoudiasl, H., Denton, P.B., Doeleman, S.S., Eichhorn, A., Eubanks, M., Fang, Y., Foschi, A., Fromm, C.M., Galison, P., Ghosh, S.G., Gold, R., Gurvits, L.I., Hadar, S., Held, A., Houston, J., Hu, Y., Johnson, M.D., Kocherlakota, P., Natarajan, P., Olivares, H., Palumbo, D., Pesce, D.W., Rajendran, S., Roy, R., Saurabh, Shao, L., Tahura, S., Tamar, A., Tiede, P., Vincent, F.H., Visinelli, L., Wang, Z., Wielgus, M., Xue, X., Yakut, K., Yang, H., Younsi, Z.: Fundamental physics opportunities with future ground-based mm/sub-mm VLBI arrays. Living Rev. Relativ. (2025). 10.1007/s41114-025-00057-0arXiv:2312.02130 [astro-ph.HE]

[13] Evans, M., Adhikari, R.X., Afle, C., Ballmer, S.W., Biscoveanu, S., Borhanian, S., Brown, D.A., Chen, Y., Eisenstein, R., Gruson, A., Gupta, A., Hall, E.D., Huxford, R., Kamai, B., Kashyap, R., Kissel, J.S., Kuns, K., Landry, P., Lenon, A., Lovelace, G., McCuller, L., Ng, K.K.Y., Nitz, A.H., Read, J., Sathyaprakash, B.S., Shoemaker, D.H., Slagmolen, B.J.J., Smith, J.R., Srivastava, V., Sun, L., Vitale, S., Weiss, R.: A horizon study for cosmic explorer: science, observatories, and community (2021). arXiv:2109.09882

[14] Evans, M., Corsi, A., Afle, C., Ananyeva, A., Arun, K.G., Ballmer, S., Bandopadhyay, A., Barsotti, L., Baryakhtar, M., Berger, E., Berti, E., Biscoveanu, S., Borhanian, S., Broekgaarden, F., Brown, D.A., Cahillane, C., Campbell, L., Chen, H.-Y., Daniel, K.J., Dhani, A., Driggers, J.C., Effler, A., Eisenstein, R., Fairhurst, S., Feicht, J., Fritschel, P., Fulda, P., Gupta, I., Hall, E.D., Hammond, G., Hannuksela, O.A., Hansen, H., Haster, C.-J., Kacanja, K., Kamai, B., Kashyap, R., Shapiro Key, J., Khadkikar, S., Kontos, A., Kuns, K., Landry, M., Landry, P., Lantz, B., Li, T.G.F., Lovelace, G., Mandic, V., Mansell, G.L., Martynov, D., McCuller, L., Miller, A.L., Nitz, A.H., Owen, B.J., Palomba, C., Read, J., Phurailatpam, H., Reddy, S., Richardson, J., Rollins, J., Romano, J.D., Sathyaprakash, B.S., Schofield, R., Shoemaker, D.H., Sigg, D., Singh, D., Slagmolen, B., Sledge, P., Smith, J., Soares-Santos, M., Strunk, A., Sun, L., Tanner, D., van Son, L.A.C., Vitale, S., Willke, B., Yamamoto, H., Zucker, M.: Cosmic explorer: a submission to the NSF MPSAC ngGW subcommittee. arXiv e-prints (2023) 10.48550/arXiv.2306.13745arXiv:2306.13745 [astro-ph.IM]

[15] Maggiore, M., Broeck, C.V.D., Bartolo, N., Belgacem, E., Bertacca, D., Bizouard, M.A., Branchesi, M., Clesse, S., Foffa, S., García-Bellido, J., Grimm, S., Harms, J., Hinderer, T., Matarrese, S., Palomba, C., Peloso, M., Ricciardone, A., Sakellariadou, M.: Science case for the Einstein Telescope. JCAP **2020**(03), 050 (2020). 10.1088/1475-7516/2020/03/050

[16] Branchesi, M., Maggiore, M., Alonso, D., Badger, C., Banerjee, B., Beirnaert, F., Belgacem, E., Bhagwat, S., Boileau, G., Borhanian, S., Brown, D.D., Leong Chan, M., Cusin, G., Danilishin, S.L., Degallaix, J., De Luca, V., Dhani, A., Dietrich, T., Dupletsa, U., Foffa, S., Franciolini, G., Freise, A., Gemme, G., Goncharov, B., Ghosh, A., Gulminelli, F., Gupta, I., Kumar Gupta, P., Harms, J., Hazra, N., Hild, S., Hinderer, T., Siong Heng, I., Iacovelli, F., Janquart, J., Janssens, K., Jenkins, A.C., Kalaghatgi, C., Koroveshi, X., Li, T.G.F., Li, Y., Loffredo, E., Maggio, E., Mancarella, M., Mapelli, M., Martinovic, K., Maselli, A., Meyers, P., Miller, A.L., Mondal, C., Muttoni, N., Narola, H., Oertel, M., Oganesyan, G., Pacilio, C., Palomba, C., Pani, P., Pasqualetti, A., Perego, A., Périgois, C., Pieroni, M., Piccinni, O.J., Puecher, A., Puppo, P., Ricciardone, A., Riotto, A., Ronchini, S., Sakellariadou, M., Samajdar, A., Santoliquido, F., Sathyaprakash, B.S., Steinlechner, J., Steinlechner, S., Utina, A., Van Den Broeck, C., Zhang, T.: Science with the Einstein Telescope: a comparison of different designs. JCAP 2023(07), 068 (2023) 10.1088/1475-7516/2023/07/068

[17] Fritschel, P., Kuns, K., Driggers, J., Effler, A., Lantz, B., Ottaway, D., Ballmer, S., Dooley, K., Adhikari, R., Evans, M., Farr, B., Gonzalez, G., Schmidt, P., Raja, S.: Report of the LSC Post-O5 Study Group. Technical Report LIGO-T2200287–v3, LIGO (2024). https://dcc.ligo.org/LIGO-T2200287/public

[18] Yunes, N., Pretorius, F.: Fundamental theoretical bias in gravitational wave astrophysics and the parameterized post-Einsteinian framework. Phys. Rev. D **80**, 122003 (2009). 10.1103/PhysRevD.80.122003. arXiv:0909.3328 [gr-qc]

[19] Bern, Z., Parra-Martinez, J., Roiban, R., Ruf, M.S., Shen, C.-H., Solon, M.P., Zeng, M.: Scattering amplitudes and conservative binary dynamics at O (G). Phys. Rev. Lett. **126**(17), 171601 (2021). 10.1103/PhysRevLett.126.171601. arXiv:2101.07254 [hep-th] 33988437 10.1103/PhysRevLett.126.171601

[20] Evstafyeva, T., Sperhake, U., Romero-Shaw, I., Agathos, M.: Gravitational-wave data analysis with high-precision numerical relativity simulations of boson star mergers (2024). arXiv:2406.02715 10.1103/PhysRevLett.133.131401 39392956

[21] Cole, P.S., Bertone, G., Coogan, A., Gaggero, D., Karydas, T., Kavanagh, B.J., Spieksma, T.F.M., Tomaselli, G.M.: Distinguishing environmental effects on binary black hole gravitational waveforms. Nature Astron. **7**, 943–950 (2023). 10.1038/s41550-023-01990-2. arXiv:2211.01362 [gr-qc]

[22] Littenberg, T.B., Cornish, N.J.: Prototype global analysis of LISA data with multiple source types. Phys. Rev. D **107**(6) (2023) 10.1103/physrevd.107.063004

[23] Dax, M., Green, S.R., Gair, J., Macke, J.H., Buonanno, A., Schölkopf, B.: Real-time gravitational wave science with neural posterior estimation. Phys. Rev. Lett. **127**(24), 241103 (2021). 10.1103/PhysRevLett.127.241103. arXiv:2106.12594 [gr-qc] 34951790 10.1103/PhysRevLett.127.241103

[24] Bhardwaj, U., Alvey, J., Miller, B.K., Nissanke, S., Weniger, C.: Sequential simulation-based inference for gravitational wave signals. Phys. Rev. D **108**(4), 042004 (2023). 10.1103/PhysRevD.108.042004. arXiv:2304.02035 [gr-qc]

